# miR-425 deficiency promotes necroptosis and dopaminergic neurodegeneration in Parkinson’s disease

**DOI:** 10.1038/s41419-019-1809-5

**Published:** 2019-08-05

**Authors:** Yong-Bo Hu, Yong-Fang Zhang, Hao Wang, Ru-Jing Ren, Hai-Lun Cui, Wan-Ying Huang, Qi Cheng, Hong-Zhuan Chen, Gang Wang

**Affiliations:** 1 Department of Neurology & Neuroscience Institute, Ruijin Hospital affiliated to Shanghai Jiao Tong University School of Medicine, 200025 Shanghai, China; 2 Department of Pharmacology and Chemical Biology, Shanghai Jiao Tong University School of Medicine, 200025 Shanghai, China; 3 School of Public health Shanghai Jiao Tong university, 200025 Shanghai, China; 4 Institute of Interdisciplinary Science, Shuguang Hospital, Shanghai University of Traditional Chinese Medicine, 201203 Shanghai, China

**Keywords:** Necroptosis, Parkinson's disease

## Abstract

A major hallmark of Parkinson’s disease (PD) is the degeneration of dopaminergic neurons in the substantia nigra, and the causative mechanism is thought to be the activation of programmed neuronal death. Necroptosis is a regulated process of cell death triggered by RIPK1. Although the pathophysiology of PD has been studied extensively, the cellular mechanism underlying dopaminergic neuron death remains unclear. In this study, we detected a specific miRNA, miR-425, in response to MPTP toxicity and dopaminergic degeneration. In MPTP-treated mice, we observed necroptosis activation and miR-425 deficiency in the substantia nigra, which is correlated with dopaminergic neuron loss. This miRNA targeted RIPK1 transcripts and promoted the phosphorylation of MLKL and necroptosis. Similarly, in the brains of PD patients, miR-425 deficiency and necroptosis activation were also confirmed in dopaminergic neuron. Furthermore, we found that genetic knockdown of miR-425 aggravated MPTP-induced motor deficits and dopaminergic neurodegeneration via early upregulation of necroptotic genes. Intracerebral miR-425 mimics (AgomiR-425) treatment attenuated necroptosis activation and dopaminergic neuron loss, and improved locomotor behaviors. In conclusion, our study suggests that miR-425 deficiency triggers necroptosis of dopaminergic neurons, and targeting miR-425 in MPTP-treated mice restored dysfunctional dopaminergic neurodegeneration and ameliorated behavioral deficits. These findings identify brain delivery of miR-425 as a potential therapeutic approach for the treatment of PD.

## Introduction

Parkinson’s disease (PD) is characterized by the degeneration of dopaminergic neurons in the substantia nigra (SN), and the causative mechanism is thought to be the activation of neuronal death^1^. Although different forms of cell death have been identified, their molecular mechanism and involvement in neurodegenerative diseases are not well elucidated^2,3^. Moreover, in PD, although the pathogenesis has been investigated extensively, the mechanism underlying dopaminergic neuron death remains unclear^4^.

Necroptosis is a regulated process of cell death triggered by receptor-interacting protein kinase 1 (RIPK1)^5,6^ and was first identified as a result of inflammation^4,7^. Pathologically, necroptosis is initiated by activation of the TNFα receptor, followed by kinase activation of RIPK1 and RIPK3^8,9^. In particular, RIPK1, a death domain-containing Ser/Thr kinase, has an established role in mediating the deleterious mechanisms downstream of type I tumor necrosis factor α receptor (TNFR1)^10^. Activated RIPK1 and RIPK3 form the necrosome complex and then recruit MLKL, leading to necroptosis execution and mitochondrial membrane disintegration^11,12^. The involvement of necroptosis is reported in neurodegenerative diseases, including amyotrophic lateral sclerosis and Alzheimer’s disease^6,13,14^.

Alterations in microRNAs (miRNAs) reportedly contribute to the pathogenic mechanisms in neurodegenerative diseases, including PD^15,16^. miRNAs are strong candidates for coordinating complex pathological processes^17^. These short noncoding RNAs act as posttranscriptional regulators of gene expression by binding to mRNA containing a miRNA recognition element. A single miRNA binding its target mRNA can orchestrate the epigenetic regulation of gene products and facilitate developmental or pathological switches, such as cell survival and death^18,19^. However, it remains unclear how miRNA might be involved in mediating necroptosis in PD.

In the present study, we hypothesized that miRNA-mediated necroptosis is involved in dopaminergic neuron death in PD. First, we confirmed whether necroptosis is activated in 1-methyl-4-phenyl-1,2,3,6-tetrahydropyridine (MPTP)-treated mice or not in order to reveal the role of miRNAs in necroptosis. Second, we investigated whether the ablation of miR-425 could aggravate pathological PD-like processes in miR-425 knockdown mice treated with MPTP. Finally, we determined whether targeting miR-425 in MPTP-treated mice could restore dysfunctional dopaminergic neurodegeneration and ameliorate the disease, thereby identifying miR-425 as a therapeutic target for PD.

## Materials and methods

### Animals and MPTP injection

C57BL/6 mice (male, 6 months old) and *Mir-425*^low^ mice (male, 3 months, 6 months, and 15 months old) used for all experiments were from Model Animal Research Center of Nanjing University. Mir-425^low^ mice were generated using CRISPR/Cas9 approach at the Nanjing Animal Center and 248 bp DNA fragment containing miR-425 was deleted to produce the null allele. Heterozygous Mir-425^low^ mice were obtained from Mir-425^low^ mice crossing with wild-type C57BL/6 mice. The primer sequences used for genotyping are as follows: forward primer: 5′-ATGGTGGCAGTCAGAGGCGA-3′; the reverse primer 5′-GTGATGATGAGAAGACCCAA-3′.

Animal experiments were performed according to the protocols and guidelines and were approved by the Ethics Committee of Shanghai Jiao Tong University School of Medicine. MPTP (30 mg/kg, Sigma-Aldrich, USA) was injected intraperitoneally in C57BL/6 mice (*N* = 5, respectively) and *Mir-425*^*low*^ or wild-type (WT) mice (*N* = 8, respectively). MPTP was injected daily for 5 days^20,21^. Mice were anesthetized with isoflurane and transcardially perfused with ice-cold phosphate-buffered saline (PBS). One half of the brain was dissected and homogenized for western blot analysis. The other half of the brain was fixed in 4% PFA overnight at 4 °C and incubated in 30% sucrose for immunostaining.

### Cell culture and transfection

Rat pheochromocytoma PC12 cells were cultured in DMEM (Gibco, USA) with 10% fetal bovine serum (FBS) at 37 °C in a 5% CO_2_ incubator. Cells were plated at a density of 10^6^ cells/cm^2^ in 6-well dishes that were coated with 100 μg/ml poly-lysine. Cell transfections were performed with AntagomiR-425-FAM, RIPK1 3′UTR or mutant plasmid (Genepharma, China) using Lipofectamine 3000 (ThermoFisher, USA). After 48 h, cells were harvested for firefly and the Renilla luciferase activities assay using the dual-luciferase reporter assay kit according to the manufacturer’s protocol (Promega, USA). The Renilla/firefly activity was used for analysis.

### Dopamine level determination

Dopamine levels were examined through high performance liquid chromatography- tandem mass spectrometry (HPLC–MS/MS)^22^. Samples were homogenized in RIPA buffer, centrifuged at 14,000 rpm for 15 min at 4 °C and analyzed for protein content by BCA protein assay reagent. Supernatant fractions were filtered, injected into an ultrasphere HPLC column and separated with a mobile phase containing 0.3 mM sodium octyl sulfate, 0.1 mM EDTA, 0.1 M sodium phosphate, and 5% (v/v) acetonitrile. With a standard curve generated with standard dopamine, the dopamine amount was then quantified.

### Behavioral tests

Motor coordination was investigated with the rotarod test. Before the experiments, animals were placed on rotating lanes for 5 min and acclimated to the testing environment. Mice were trained for 2 min at a fixed speed of 4 rpm. After training, mice were performed four trials for 60 s with programmed acceleration speed starting from 4 to 40 rpm. The time of falling off the rotating rod was recorded. Spontaneous locomotor activity was examined in the open field test. The mice were individually placed into the center of an open field box (38 × 38 cm) in a noise and light-controlled room. The spontaneous locomotor activities (central-area distance and whole-area distance) of each mouse were recorded and analyzed in 300 s^23,24^. The parameters were analyzed by the SuperMaze tracking system (Shanghai, China).

### The enzyme-linked immunosorbent assay (ELISA)

Cell culture media were collected 72 h after transfection and cell debris was removed by centrifugation. For brain lysates, mouse brains were homogenized and diluted with PBS. Mouse TNFα was detected using sandwich ELISA kits (ThermoFisher, USA) following the manufacturer’s instructions. Plates were read at 450 nm on a Synergy MX plate reader (BioTeck, USA).

### Stereotaxic injection

AgomiR-425 with FAM labeling (Genepharma, China) was injected into the SNpc of mice brain. Six-month-old mice from each group were anesthetized with isoflurane. Intracerebral injection was performed with following coordinates: −2.8 mm anteroposterior, −1.2 mm mediolateral, and −4.3 mm dorsoventral. Five microliters of AgomiR-425 suspension was injected into each site using a 10 μl Hamilton syringe over a 5-min period. The needle remained in place for 5 min after complete injection then slowly removed. The mice were placed on a pad until recovery from the anesthesia.

### Immunostaining and quantification

Harvested mouse brain tissues were fixed in 4% paraformaldehyde (PFA) and embedded in paraffin or OTC (SAKURA, USA). Specimen was cut to 4-μm-Paraffin-embedded brain sections or 40-μm-free-floating mouse brain sections. Sections were washed and blocked in 5% BSA, 0.3% Triton X-100 for 30 min and incubated overnight with anti-TH (1:500, Abcam, USA) antibody, anti-RIPK1 antibody (1:1,000, Abcam, USA,), anti-RIPK3 (1: 1000, Abcam, USA,), or anti-pMLKL (1:500, Abgent, China) at 4 °C. The slides were washed three times in PBST and incubated with AlexaFluor 488-conjugated donkey anti-rabbit or AlexaFluor 594 anti-mouse IgG secondary antibodies (Invitrogen), and image were acquired using a confocal microscope (Zeiss, Germany). Mouse miR-425 in situ hybridization (ISH) was performed on paraffin-embedded brain sections using a microRNA ISH buffer set and a miRCURY LNA miR-425 probe (Exiqon, Denmark) according to the manufacturer’s instructions.

### Stereological estimation of TH-positive neurons

To estimate the number of nigral dopaminergic neurons, stereological counts were performed and every sixth section was selected between levels 2.80 and 3.80 mm from the bregma. After delineation of the SN pars compacta with a ×4 objective, counts were performed at ×60 magnification in ImageJ with the following parameters: 8 µm height of an optical disector, 50 × 50 μm counting frame, 100 × 100 μm area of a grid. Coefficient of error <0.10 were accepted.

### Transmission electron microscopy

PC12 cells were collected, fixed with 2.5% glutaraldehyde for 2 h, and embedded in Epon resin after dehydration. The ultrastructure of mitochondria was obtained from ultrathin sections with a CCD camera of a Hitachi transmission electron microscope at an accelerating voltage of 80 kV.

### Western blot analysis

Brain tissue or cells were lysed with lysis buffer and subjected to a 12,000 rpm centrifugation. Total protein was determined using the BCA Protein Assay Reagent (ThermoFisher, USA). Fifty micrograms of protein and sample buffer was loaded onto 10% SDS-PAGE gels, and then the gels were transferred to PVDF membranes. Membranes were blocked and then incubated with primary antibodies anti-RIPK1 antibody (1:1,000), anti-RIPK3(1:1,000), anti-MLKL (1:1,000), or anti-pMLKL antibody (1:500) overnight at 4 °C. After washing three times with TBST, membranes were incubated with secondary peroxidase-conjugated antibodies, and protein blots were visualized using the ECL kit. GAPDH was used as a loading control. Images were captured, and band intensities were quantified using an Odyssey Image Station (LI-COR, USA).

### RNA sequencing and bioinformatics analysis

Total RNA was extracted using the RNeasy Mini Kit (QIAGEN, Germany), and RNA-seq libraries were constructed per the Illumina TrueSeq RNA sample preparation kit. High-throughput sequencing was performed using the Illumina HiSeq 4000 (Aksomics, China). Differentially expressed miRNAs were analyzed and plotted in heatmap in R software. To explore gene changes in SNpc after MPTP treatment, publicly available GEO data sets GSE17542, GSE47788, GSE60080, and GSE7707 were used for bioinformatics analysis. Differentially expressed genes were analyzed and plotted in a volcano plot in R software.

### Real-time qPCR

Total RNA was extracted using the RNeasy Mini Kit (QIAGEN, Germany), and cDNA was synthesized with a cDNA reverse transcription kit (Takara, Japan). Real-time qPCR was performed with a LightCycler 480 instrument with SYBR Green reagents (Takara, Japan).

### Human brain material

Postmortem brain tissues from the midbrain of four control and four PD patients with clinical diagnosis and neuropathological confirmation were obtained from the Human Brain Bank of Peking Union Medical College (PUMC). Written informed consent for the use of brain tissues and clinical data for research purposes was obtained and human brain tissues were analyzed anonymously. This study was approved by ethical committees of Shanghai Jiao-Tong University.

### Reactive oxygen species (ROS) detection

Cellular oxidative stress production was investigated through the Cellular ROS/Superoxide detection assay kit (ab139476, Abcam, UK) following the manufacturer’s protocol. Briefly, cells were seeded onto 96-well black/clear bottom plates and transfected with AntagomiR-425 or scramble. After 48 h, cells were stained with an oxidative stress reagent. Fluorescence was then measured using a fluorescent microplate reader (Biotek Synergy MX, USA).

### Statistical analysis

GraphPad Prism 7.0 software was used to analyze the data, which are reported as the means ± SEM. Unpaired Student’s *t* test was used for comparison between two groups. For more than two groups, one-way or two-way ANOVA followed by post hoc Dunnett’s test was applied. Statistical significance was defined as *P* < 0.05.

## Results

### Cellular localization and RIPK1 upregulation in the SN in the MPTP mouse model

**Fig. 1:**
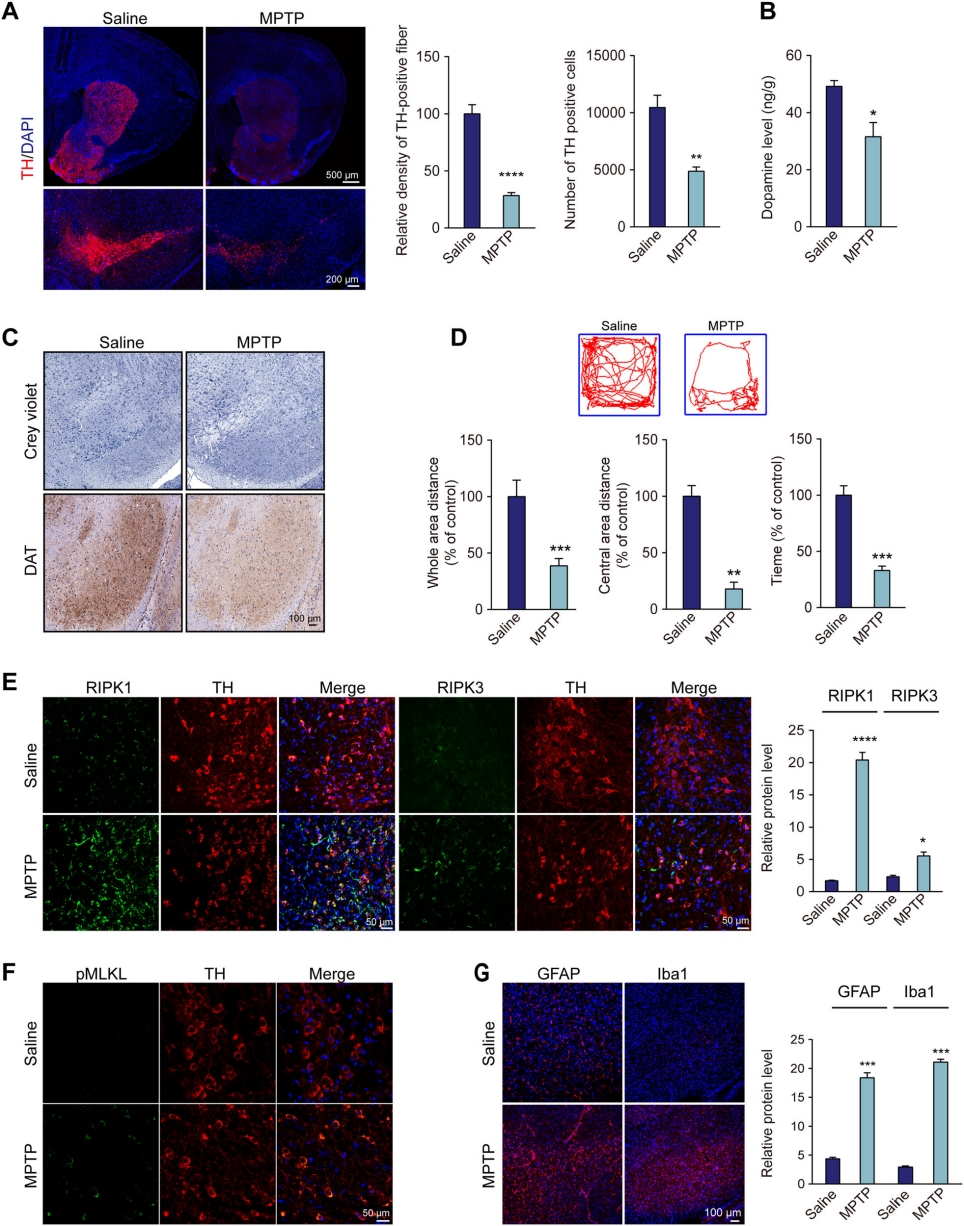
Cellular localization and RIPK1 upregulation in the SNpc in the MPTP mouse model. **a** Immunofluorescence for TH in the striatum (upper panel) and SNpc (lower panel) of MPTP mice. Quantification of TH-positive neuronal fibers in the striatum and TH-positive neurons in the SNpc. **b** Quantification of dopamine in the striatum by HPLC. **c** Immunohistochemistry for cresyl violet-positive cells and dopamine transporter (DAT) in the SNpc. **d** Motor behavior in the open field in the MPTP-induced mouse model. Representative tracks of mice in the open field chamber over 5 min. Whole-area distance and central-area distance were measured. Motor behavior on the rotarod tests in the MPTP-induced mouse model. Time spent on the rotarod was measured. **e** Representative images and quantification of immunofluorescence for RIPK1 and RIPK3 in dopaminergic neurons. **f** Immunohistochemistry for phosphorylated MLKL (pMLKL) in dopaminergic neurons. **g** Representative confocal micrographs of Iba1 and GFAP staining in the SNpc. All data represent the mean ± SEM. Student’s *t* test, *P < 0.05, **P < 0.01, ***P < 0.001, and ****P < 0.0001

To confirm MPTP-induced dopaminergic degeneration, we first assessed PD-like pathology and motor dysfunctions in C57BL/6 mice after MPTP treatment (30 mg/kg) for 5 consecutive days. As expected, both the significant degeneration of tyrosine hydroxylase (TH)-positive neurons and the loss of cresyl violet-stained neurons were observed in the substantia nigra par compacta (SNpc) (Fig. 1a). Levels of striatal dopamine in the MPTP group were remarkably decreased compared with those in the control group via HPLC analysis (Fig. 1b). Consistent with the neuropathological and biochemical findings, motor dysfunctions were observed following MPTP treatment, and MPTP-treated mice showed a significant decrease in overall motor activity with less mobile time in the open field test, as well as impaired balance and coordination with an apparent reduction in movement time on the rotarod test. Collectively, MPTP-treated mice successfully exhibited PD-like pathology and an impaired locomotor phenotype (Fig. 1a–d).

A recent report found that administration of necrostatin-1 (Nec-1), a pharmacological inhibitor of necroptosis, effectively attenuated MPTP-induced dopaminergic neuron loss and mitochondrial toxicity, suggesting that necroptosis existed following MPTP treatment^25^. However, the roles of three key proteins, RIPK1, RIPK3, and MLKL, and local neuroinflammation involved in necroptosis remain unclear and need to be further investigated. In the present study, following MPTP treatment for 5 consecutive days, immunofluorescence results revealed that RIPK1 and RIPK3 were increased in TH-positive neurons in the mouse SN (Fig. 1e). This result suggested that necrosomes were formed as a key initiator of necroptosis. Noticeably, RIPK1 and pMLKL levels were significantly higher in the MPTP-treated group (Fig. 1e, f and Fig. S2A, B). Considering the beneficial effects of Nec-1 treatment targeting RIPK1 and the significant changes in RIPK1 expression in the SNpc of the MPTP mouse model, we believe that RIPK1 plays a more critical role in inducing necroptosis. With regard to RIPK1, it was reported that it can trigger both necroptosis and apoptosis^26,27^, we further investigated the role of necroptosis and apoptosis in the context of dopaminergic neurodegeneration. We found that even though apoptotic marker cleaved Caspase-3 immunoreactivity was observed in MPTP-treated mice, however, cleaved Caspase-3 was rarely colocalized with TH-positive neuron. In contrast, necroptotic marker pMLKL was mostly colocalized with TH-positive neuron. This result confirmed that necroptosis played a major role in the execution of dopaminergic neuron loss. Moreover, activation of microglia and astroglia was observed in the SNpc with increased TNFα release (Fig. 1g and Fig. S2C). Taken together, these data indicate that activated necroptosis and the inflammatory response occurred in degenerated dopaminergic neurons in the MPTP mouse model, correlating with an upregulation of RIPK1.

### miR-425 was correlated with RIPK1 expression and MPTP-induced dopaminergic degeneration

**Fig. 2:**
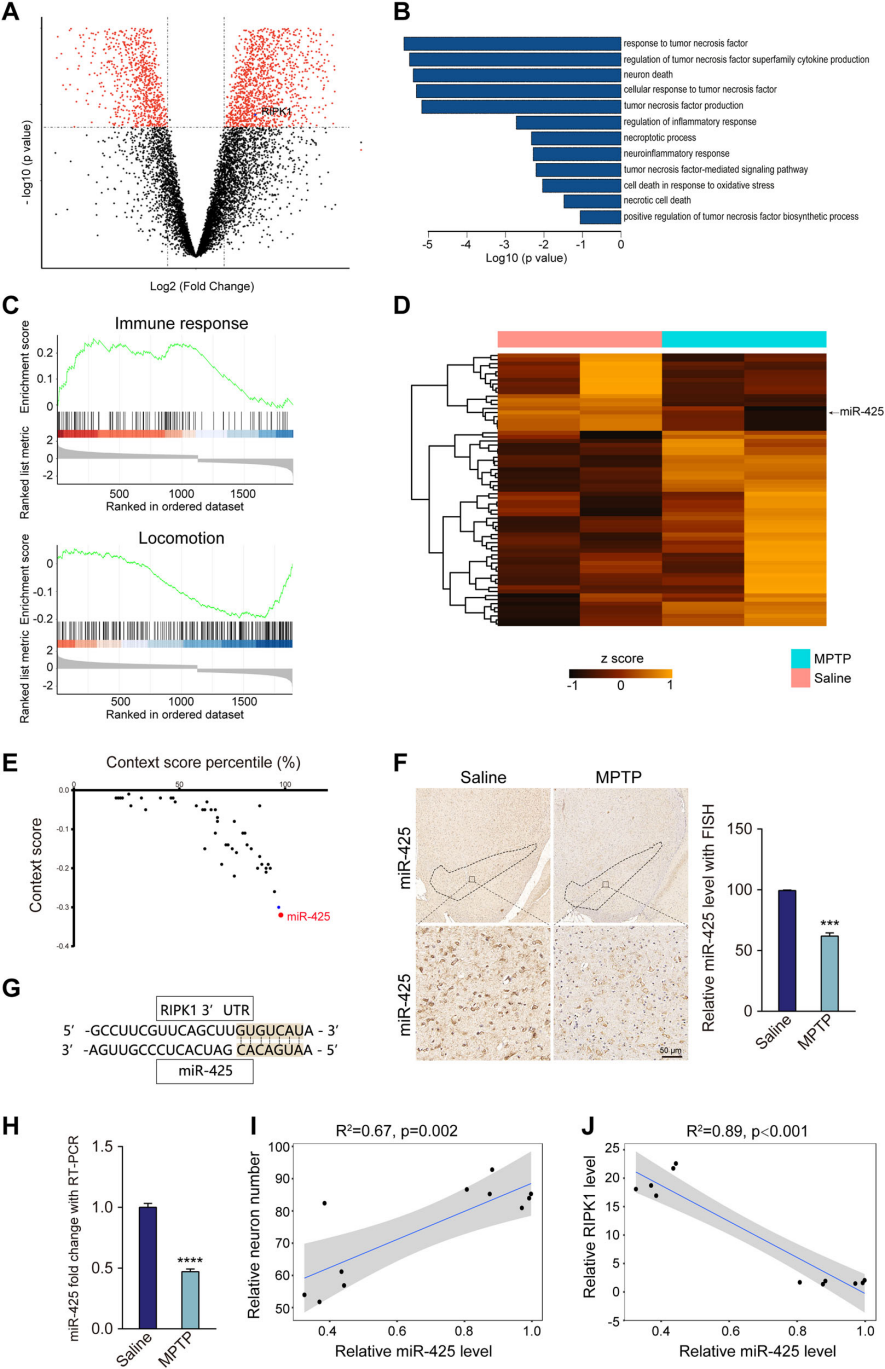
miR-425 was correlated with RIPK1 expression and MPTP-induced dopaminergic degeneration. **a** Volcano plots showing the mRNA expression profile of the MPTP mouse model compared with the saline control mice. **b** Summary of the gene ontology terms of differentially expressed genes in the MPTP mouse model. **c** Gene set enrichment analysis of differentially expressed genes in the MPTP mouse model. The enrichment plots of the hallmark of immune response and locomotion are shown. **d** Expression heatmap of differential miRNA profiles in the SNpc of the MPTP mouse model and saline control mice. **e** Context score of predicted miRNAs associated with RIPK1. **f** miR-425 expression in mouse brains detected using chromogenic ISH. **g** Seed region of miR-425 in the 3′UTR of mouse RIPK1. **h** Quantification of miR-425 levels in mouse midbrains by RT-PCR. **i** Correlations of miR-425 expression with cresyl violet-positive neurons in the SNpc in both MPTP and saline groups. **j** Correlations of the expression of miR-425 with that of RIPK1 in the SNpc in both MPTP and saline groups. All data represent the mean ± SEM. Student’s *t* test, ***P < 0.001 and ****P < 0.0001

To further investigate MPTP-induced necroptosis-associated gene alterations, gene profiles of the SNpc using an mRNA microarray were analyzed. A volcano plot of gene expression indicated that RIPK1 was significantly increased after MPTP treatment (Fig. 2a). Given the significant number of genes that differentially changed, gene ontology (GO) and gene set enrichment analysis (GSEA) were adopted to identify the MPTP-associated pathways. GO analysis showed that TNFα response and regulation, neuronal death, and neuroinflammatory responses were associated with MPTP toxicity (Fig. 2b). Moreover, the GSEA results indicated that the immune response-related gene set was significantly activated, whereas the locomotion-related gene set was suppressed (Fig. 2c), consistent with previous findings in an MPTP mouse model^28,29^.

To explore the possible mechanism underlying necroptosis in MPTP-induced Parkinsonism, we speculated that MPTP regulated necroptosis-associated gene expression through posttranslational modification. As miRNAs are the best known to exert posttranslational control, we first screened miRNAs involved in RIPK1 regulation. Using miRNA sequencing (miRNA-seq) of SNpc tissue from MPTP- and saline-treated mice, we identified the most significantly changed miRNA in the SNpc after MPTP treatment (Fig. 2d). Furthermore, we chose RIPK1 as the target gene to screen miRNA binding the 3′UTR of RIPK1 mRNA using online prediction programs, including miRbase and Targetscan (www.mirbase.org and www.targetscan.org). Finally, we identified 52 miRNAs and, using established programs, revealed that miR-425 is a possible candidate with a higher context score and percentile compared with other dysregulated miRNAs (Fig. 2e, g and Table S1)^30^.

Using ISH and RT-PCR, we confirmed that miR-425 was reduced in the SNpc of MPTP-treated mice (Fig. 2f–h). Moreover, miR-425 deficiency was correlated with decreased RIPK1 expression and dopaminergic neuron loss (Fig. 2i, j). These results suggest that miR-425 may be involved in dopaminergic pathology by regulating RIPK1.

### miR-425 promoted necroptosis by targeting RIPK1

**Fig. 3:**
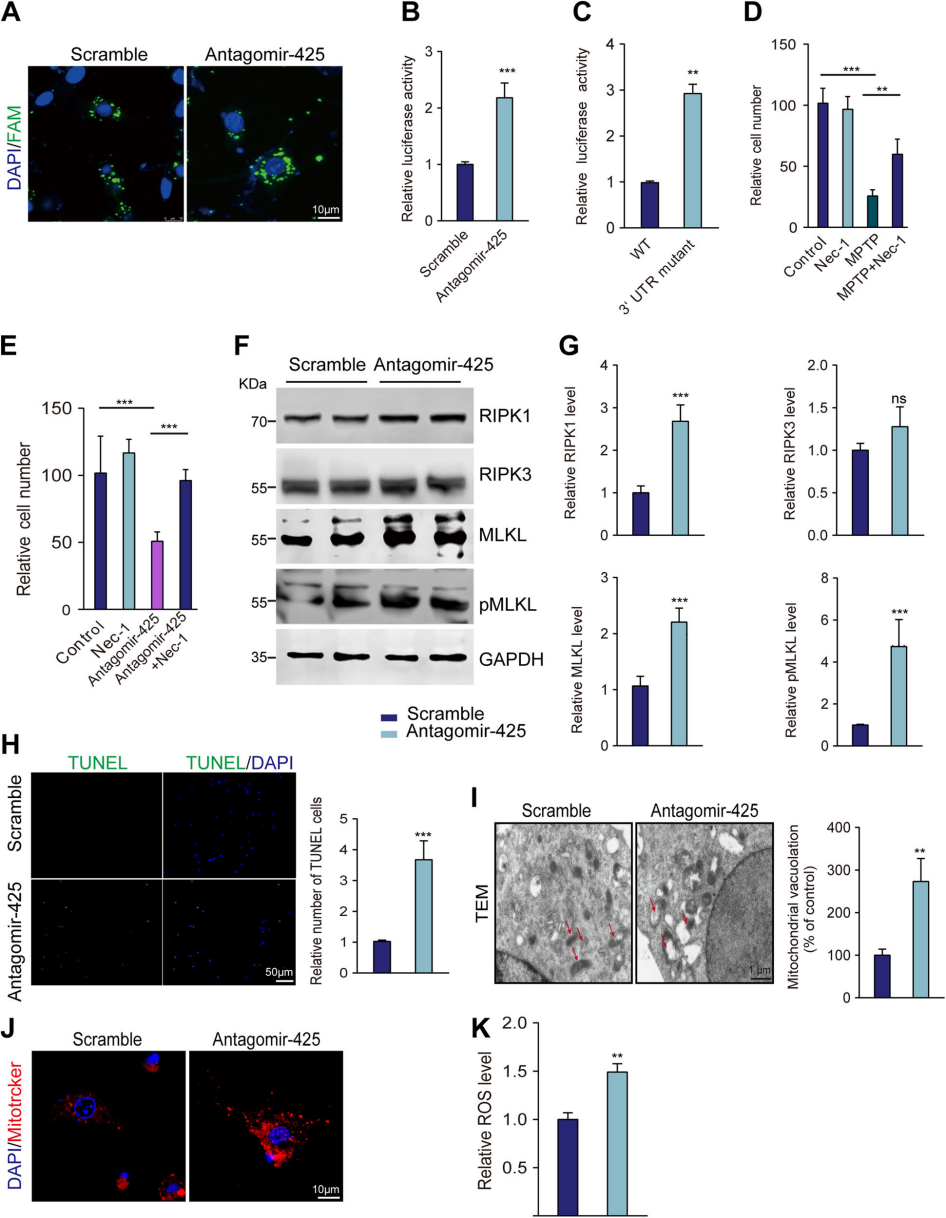
miR-425 promoted necroptosis by targeting RIPK1. **a** FAM immunofluorescence tracing of transfected AntagomiR-425 and scrambled control. **b** Luciferase activity of PC12 cells cotransfected with the WT 3′UTR of RIPK1 luciferase reporter plasmids together with AntagomiR-425 and scramble control. **c** Luciferase activity of PC12 cells cotransfected with the WT or mutant 3′UTR of RIPK1 luciferase reporter plasmids together with AntagomiR-425. **d** Quantification of PC12 cells 3 days after treatment with MPTP, Nec-1, or vehicle control. **e** Quantification of PC12 cells 3 days after treatment with AntagomiR-425, Nec-1, or vehicle control. **f** Immunoblotting of RIPK1, RIPK3, MLKL, and pMLKL expression in PC12 cells transfected with AntagomiR-425 or scrambled control. **g** Quantification of RIPK1, RIPK3, MLKL, and pMLKL expression in PC12 cells transfected with AntagomiR-425 or scrambled control. **h** TUNEL assay of PC12 cells 3 days after treatment with AntagomiR-425. **i** Representative mitochondria are shown using TEM and quantification of mitochondria vacuolation. **j** Representative mitochondria are shown using MitoTracker Red staining. **k** ROS assay of PC12 cells 3 days after treatment with AntagomiR-425. All data represent the mean ± SEM. In **d**, **e**, one-way ANOVA followed by Dunnett’s test was applied. Other experiments used Student’s *t* test, **P < 0.01, ***P < 0.001, and ns, not significant

To further ascertain the relationship between miR-425 and RIPK1, we transfected a synthetic miR-425 inhibitor, AntagomiR-425, into PC12 cells to mimic miR-425 deficiency observed in the SNpc of the MPTP mouse model (Fig. 3a). A luciferase reporter assay was performed using this cell model to examine the specificity of miR-425 targeting RIPK1 mRNA. We found that miR-425 inhibition promoted luciferase activity, moreover, mutant RIPK1 3′UTR interrupted the binding of miR-425 with RIPK1 mRNA, resulting in increased luciferase activity in contrast to the wild-type (WT) RIPK1 3′UTR (Fig. 3b, c).

To better understand the contribution of miR-425 inhibition in necroptosis, after transfecting the miR-425 inhibitor AntagomiR-425 in PC12 cells, we found that inhibition of miR-425 suppressed neuron viability and promoted cell death. However, these effects were reversed by the necroptosis inhibitor Nec-1 (Fig. 3d, e). Western blotting revealed that necroptosis-associated proteins, including RIPK1, MLKL, and pMLKL, were significantly increased following AntagomiR-425 transfection in PC12 cells (Fig. 3f, g). In addition, the TUNEL assay revealed that miR-425 inhibition promoted neuron death relative to that in cells transfected with the scramble control (Fig. 3h). Thus, these observed findings suggest that miR-425 inhibition promotes necroptosis.

Previous studies indicated that RIPK1 was a trigger for mitophagy and ROS production^11^. After confirming the relationship miRNA between miR-425 and RIPK1, we next sought to ascertain whether miR-425 deficiency leads to mitochondrial dysfunction. Structure analysis of mitochondria using transmission electron microscopy (TEM) confirmed that the length of mitochondria was decreased and presented a rounded morphology following miR-425 inhibition (Fig. 3i, j). MitoTracker staining revealed that miR-425 inhibition promoted mitochondrial accumulation and fractions in the cell body accompanied by increased ROS production (Fig. 3j, k). The alterations in mitochondrial dysfunction following miR-425 inhibition were very similar to the changes in MPTP-induced mitochondrial toxicity as previously reported^31^. Given these results, we believe that the miR-425-RIPK1 pathway plays a critical role in the execution of necroptosis and dopaminergic degeneration.

### miR-425 deficiency and necroptosis activation were observed in human PD brains

**Fig. 4:**
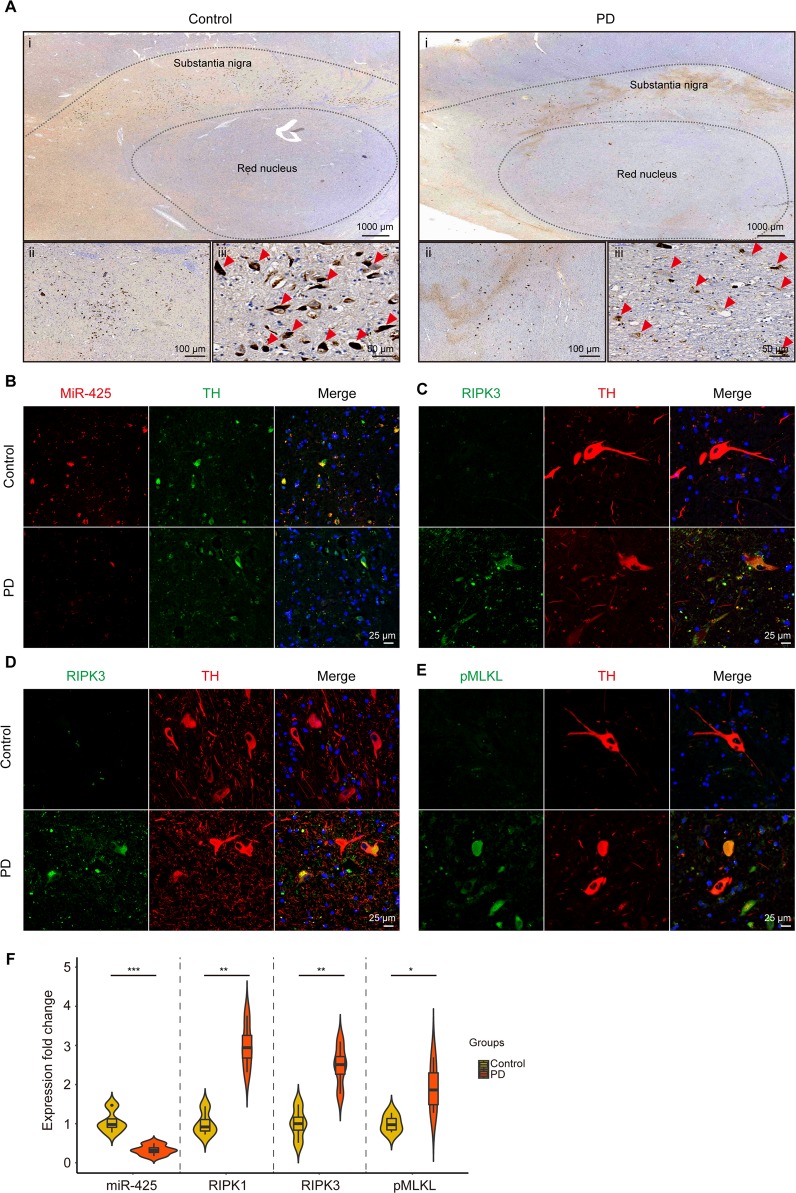
miR-425 deficiency and necroptosis activation were observed in human PD brains. **a** Chromogenic ISH of miR-425 expression in substantia nigra of PD brains and control; **b** Fluorescence ISH of miR-425 and colocalization with dopaminergic neurons in substantia nigra of PD brains and control; **c**–**e** Immunohistochemistry for RIPK1, RIPK3 and pMLKL in dopaminergic neurons in substantia nigra of PD brains and control; **f** Violin plots of miR-425, RIPK1, RIPK3, and pMLKL expression in substantia nigra of PD brains and control. Box plot with bars represent the mean ± SEM. Student’s *t* test, *P < 0.05, **P < 0.01, and ***P < 0.001

To further confirm the role of miR-425 and necroptosis in dopaminergic neurodegeneration, we validated the changes of miR-425 and necroptotic markers in SN of patients with PD. The results showed that miR-425 was markedly reduced in SN of PD brains (Fig. 4a). Moreover, confocal imaging indicated a higher degree of colocalization between miR-425 and dopaminergic neurons (Fig. 4b). Immunofluorescence staining revealed a higher RIPK1 immunoreactivity in dopaminergic neurons of PD cases compared with the control. Similarly, we found a significantly increase in RIPK3 and pMLKL in dopaminergic neurons of PD brains (Fig. 4c–f). Thus, those data indicated that miR-425 deficiency was involved in dopaminergic neurodegeneration by triggering necroptosis activation in SN of PD patients.

### miR-425 knockdown exacerbated MPTP-induced dopaminergic neuron loss and impaired locomotor behaviors

**Fig. 5:**
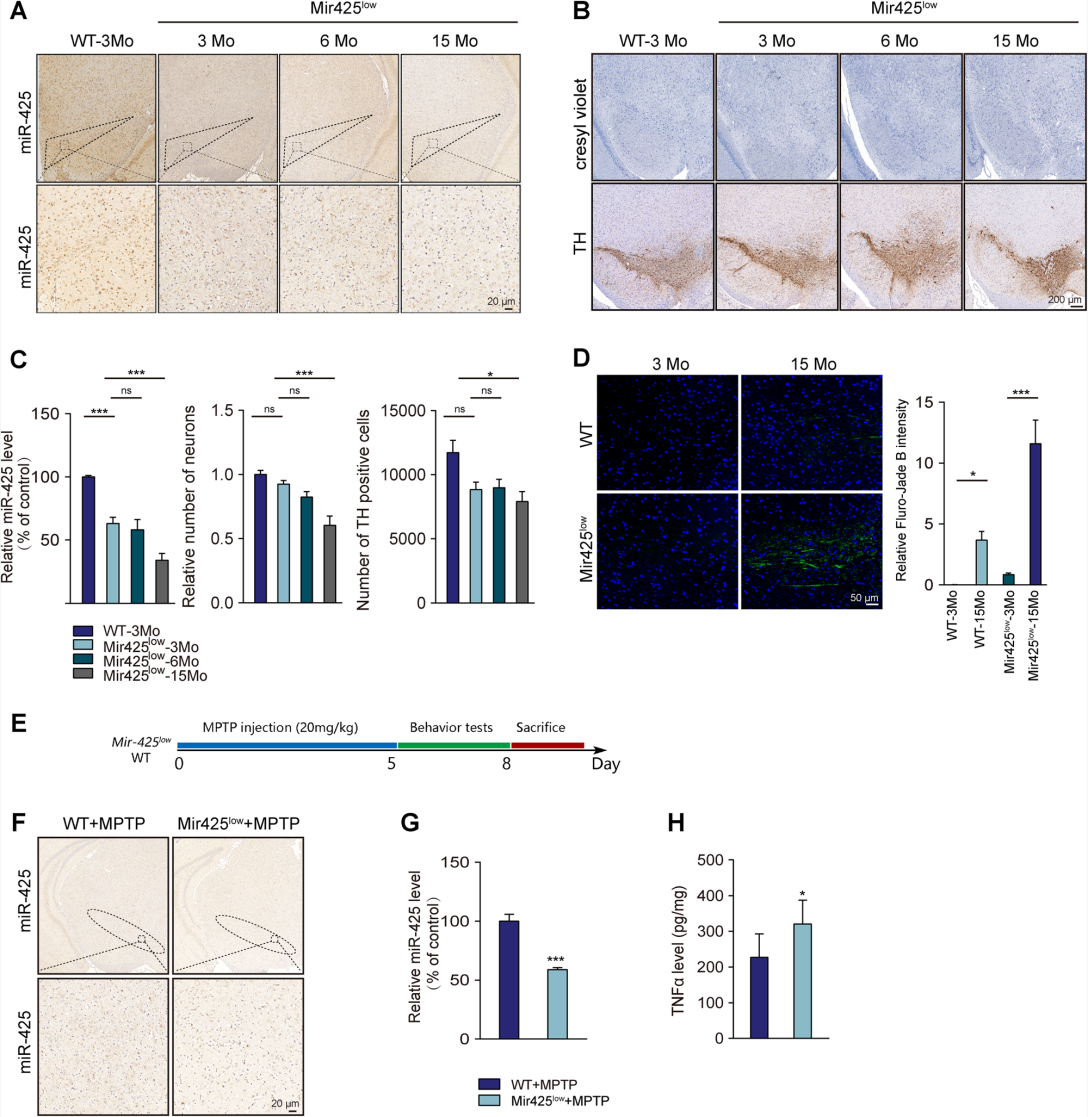
miR-425 knockdown induced age-dependent dopaminergic degeneration. **a** Chromogenic ISH of miR-425 expression in *Mir-425*^*low*^ mice at 3 months (3 Mo), 6 months (6 Mo), 15 months (15 Mo) and in WT mice. **b** Immunohistochemistry for TH and cresyl violet-positive cells in *Mir-425*^*low*^ mice at 3 months, 6 months, 15 months and in WT mice. **c** Quantification of miR-425 level, TH-positive neuronal fibers in the striatum and TH-positive neurons in the SNpc. **d** Fluro-Jade B staining in SNpc of *Mir-425*^*low*^ mice and WT mice at 3 months and 15 months. **e** Study design of MPTP-treated *Mir-425*^*low*^ mice and WT control mice. **f** Chromogenic ISH of miR-425 expression of MPTP-treated *Mir-425*^*low*^ mice and WT control mice. **g** Quantification of miR-425 expression in MPTP-treated *Mir-425*^*low*^ mice and WT control mice. **h** Quantification of TNFα levels in MPTP-treated *Mir-425*^*low*^ mice and WT control mice using ELISA. All data represent the mean ± SEM. Two-way ANOVA followed by post hoc Dunnett’s test was applied, *P < 0.05 and ***P < 0.001, ns, not significant

To address the relationship between miR-425 deficiency and necroptosis in vivo, we generated heterozygous *Mir-425*^*low*^ mice with a C57BL/6 background. First, miR-425 ISH confirmed that *Mir-425*^*low*^ mice showed decreased levels of miR-425 in the SNpc compared with those in WT mice at 3 months old (Fig. 5a). Moreover, we found that miR-425 levels in the SNpc were further decreased at 15 months of age in *Mir-425*^*low*^ mice (Fig. 5a and Fig. S3). To determine the effects of miR-425 knockdown on necroptosis and neuron loss, the findings indicated that TH-positive and cresyl violet-positive neuron loss was not detected at 3 or 6 months of age but was observed at 15 months (Fig. 5b, c). However, in WT mice we could not observe such age-associated changes of miR-425 and dopaminergic neuron in SNpc (Fig. S2). To validate the results, Fluro-Jade B staining was performed to show degenerative neurons in the brain (Fig. 5d). As expected, *Mir-425*^*low*^ mice at 15 months of age had more Fluro-Jade B positive neurons in the SNpc than 3-month *Mir-425*^*low*^ mice did. Taken together, these results suggest that aging is a critical risk factor for dopaminergic degeneration and is also involved in triggering miR-425 deficiency in the SNpc of mice.

**Fig. 6:**
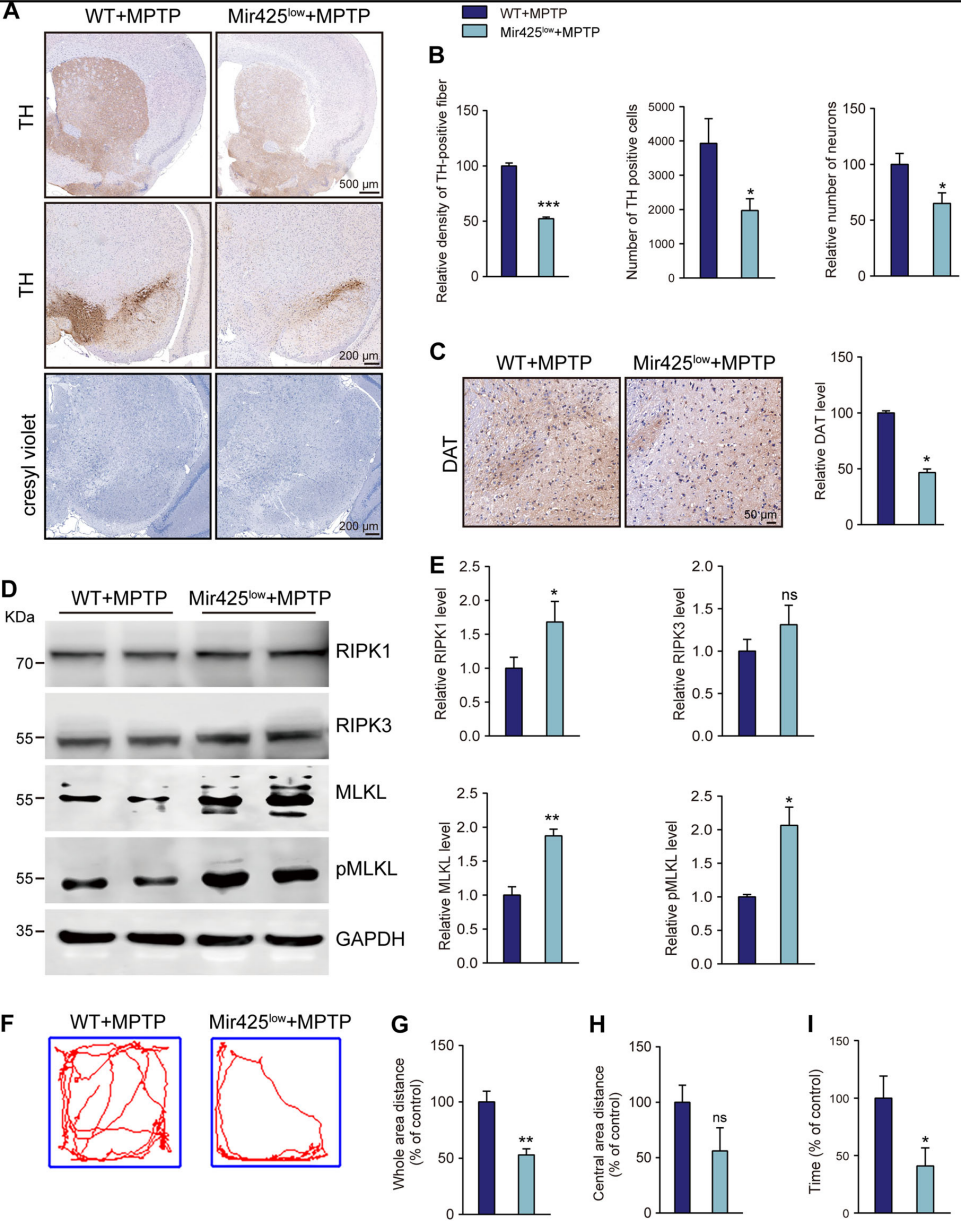
miR-425 knockdown exacerbated MPTP-induced dopaminergic neuron loss and impaired locomotor behaviors. **a** Immunohistochemistry for TH in the striatum and SN and cresyl violet-positive cells of MPTP-treated *Mir-425*^*low*^ mice. **b** Quantification of TH-positive neuronal fibers in the striatum and TH-positive neurons and cresyl violet-positive cells in the SNpc. **c** Immunohistochemistry and quantification of dopamine transporter (DAT) in the SNpc. **d** Immunoblotting of RIPK1, RIPK3, MLKL, and pMLKL expression in the SNpc of MPTP-treated *Mir-425*^*low*^ and WT mice. **e** Quantification of RIPK1, RIPK3, MLKL, and pMLKL expression in the midbrain of MPTP-treated *Mir-425*^*low*^ and WT mice. **f** Representative tracks of mice in the open field chamber over 5 min. **g** Locomotor behavior in the open field in MPTP-treated *Mir-425*^*low*^ and WT mice, whole-area distance and central-area distance were measured. **i** Time spent on the rotarod for MPTP-treated *Mir-425*^*low*^ and WT mice was measured. All data represent the mean ± SEM. Student’s *t* test, *P < 0.05, **P < 0.01, ***P < 0.001, and ns, not significant

To further investigate the contribution of miR-425-mediated necroptosis in MPTP-induced dopaminergic neurodegeneration and rule out possible involvement of aging, we sought to introduce MPTP-treated *Mir-425*^*low*^ mice and chose 6-month-old mice for injection (Fig. 5e). Compared with the WT mice, *Mir-425*^*low*^ mice showed more severe miR-425 deficiency and inflammatory cytokine TNFα release in the brains of mice (Fig. 5f–h). Moreover, miR-425 knockdown mice showed fewer TH neurons, a decreased density of cresyl violet-stained cells and fewer DAT-positive neurons relative to the WT mice after MPTP treatment (Fig. 6a–c). In all, miR-425 knockdown aggravated dopaminergic degeneration pathology in MPTP-treated mice.

To investigate the regulation of necroptosis in MPTP-treated *Mir-425*^*low*^ mice, western blotting revealed that RIPK1 as well as MLKL and pMLKL expression was significantly increased in the SNpc of *Mir-425*^*low*^ mice (Fig. 6d, e). These results suggested that *Mir-425*^*low*^ mice showed more highly activated necroptosis in dopaminergic neurons following MPTP treatment, resulting in more severe degenerative pathology.

To determine the behavioral changes of miR-425 knockdown in MPTP-treated mice, the open field test and rotarod test were used. miR-425 knockdown mice exhibited less mobile time with decreased motor activity in the open field test and displayed shorter coordination time in the rotarod test (Fig. 6f–i). Together, miR-425 knockdown mice showed more severe dopaminergic degeneration pathology and motor dysfunction after MPTP treatment.

### AgomiR-425 treatment reduced MPTP-induced necroptosis and restores behavioral deficits

**Fig. 7:**
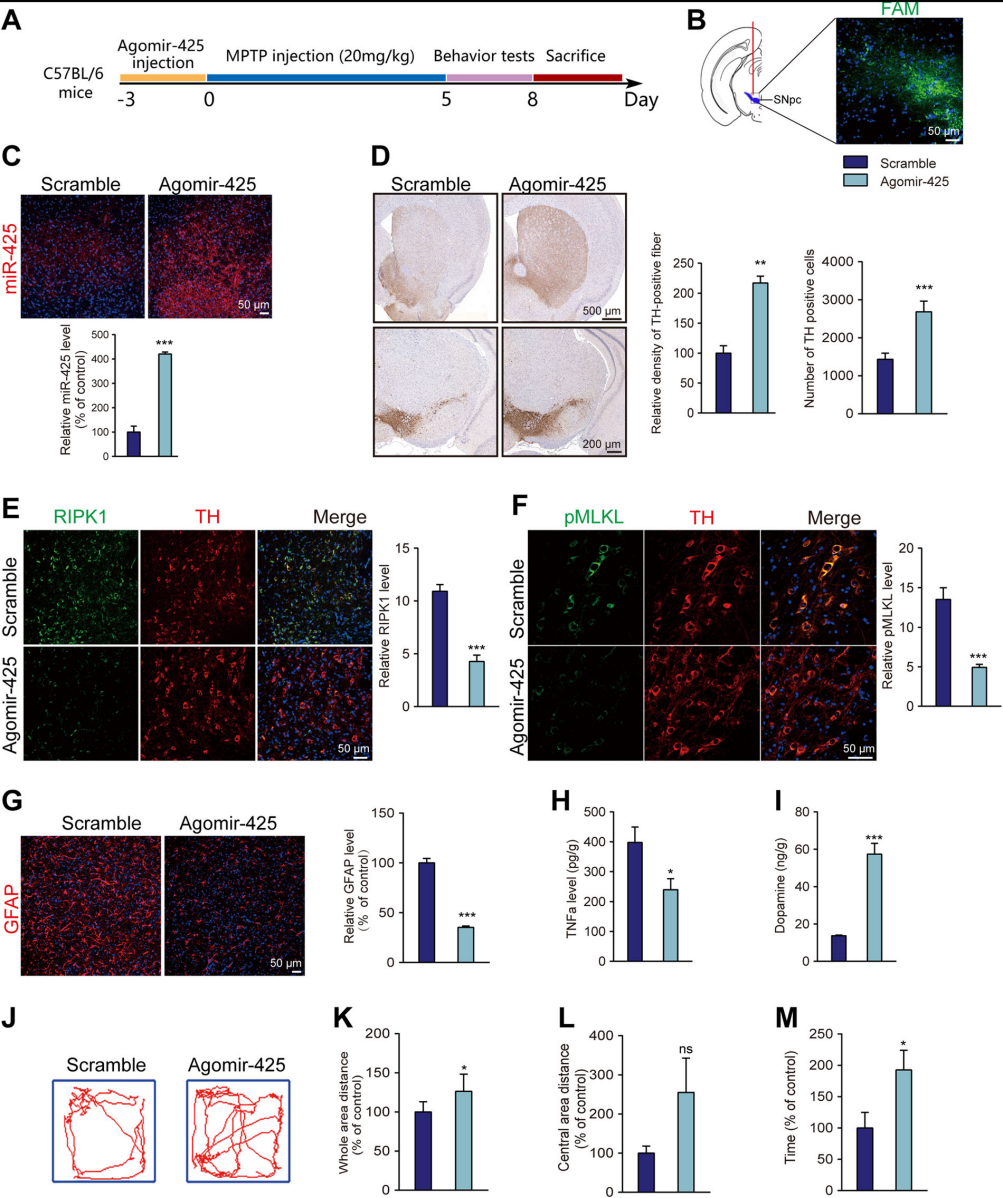
AgomiR-425 treatment reduced MPTP-induced necroptosis and behavioral deficits. **a** Study design of miR-425 intracranial injection in an MPTP mouse model. **b** FAM immunofluorescence tracing of injected AgomiR-425 in the SNpc of mice. **c** Fluorescence ISH of miR-425 expression after AgomiR-425 or scramble control injection in the SNpc of mice. **d** Immunohistochemistry for TH in the striatum and SN of AgomiR-425-injected mice and scramble-injected mice. Quantification of TH-positive neuronal fibers in the striatum and TH-positive neurons in the SNpc. **e** Representative images and quantification of immunohistochemistry for RIPK1 in dopaminergic neurons. **f** Representative images and quantification of immunohistochemistry for pMLKL in dopaminergic neurons. **g** Representative confocal micrographs and quantification of GFAP staining in the SNpc. **h** Quantification of TNFα level using ELISA. **i** Quantification of dopamine by HPLC in the striatum. **j** Representative tracks of mice in the open field chamber over 5 min. **k**, **l** Central-area distance and whole-area distance were measured. **m** Time spent on the rotarod for MPTP-induced *Mir-425*^*low*^ and WT mice was measured. All data represent the mean ± SEM. Student’s *t* test, *P < 0.05, **P < 0.01, ***P < 0.001, and ns, not significant

After demonstrating the role of miR-425 in MPTP-induced necroptosis, we investigated whether miR-425 supplementation in dopaminergic neurons could ameliorate PD-like pathology and motor dysfunction. WT mice received a stereotactic injection of miR-425 mimics (AgomiR-425) into both sides of the SNpc and were administered MPTP for 5 days (Fig. 7a). First, we confirmed the successful transfection at the site of the the SNpc by tracing fluorescence-labeled miRNA (Fig. 7b).

Immunofluorescence of miR-425 ISH confirmed that miR-425 was significantly increased following AgomiR-425 injection (Fig. 7c). To explore the effects of miR-425 supplementation on dopaminergic necroptosis, the results revealed that AgomiR-425 injection led to significant preservation of TH-positive fibers and neurons in the striatum and SNpc (Fig. 7d). Importantly, we found that AgomiR-425 specifically decreased RIPK1 expression and protected TH-positive neurons in the SNpc (Fig. 7e). Meanwhile, the level of MLKL phosphorylation was decreased in the SNpc after AgomiR-425 treatment (Fig. 7f), and TNFα levels were also decreased with reduced glial activation (Fig. 7g, h). Noticeably, dopamine levels were increased in the striatum (Fig. 7i), suggesting that the dopaminergic system was relatively protected from MPTP toxicity with AgomiR-425 treatment.

In addition, AgomiR-425 attenuated locomotor impairments by MPTP. In the open field test, AgomiR-425 injection increased the overall motor activity with more mobile time (Fig. 7j–l). In the rotarod test, AgomiR-425-treated mice showed better balance and coordination with increased movement time (Fig. 7m).

## Discussion

Progressive loss of dopaminergic neuron in the SN is a cardinal feature of PD. However, the precise molecular mechanism by which neuron death occurs remains to be elucidated. Revealing the mechanisms leading to neuronal loss is essential to develop new therapeutic strategies to delay or reverse the progression of PD^32^. A growing body of evidence suggests that the regulation of dopaminergic neurodegeneration is critical to reveal the pathogenesis of PD^2,33,34^. Here, we confirmed that necroptotic processes are involved in the neurodegeneration of dopaminergic neurons via miR-425-mediated RIPK1 activation. Firstly, in this study, we identify that miR-425 deficiency is associated with dopaminergic neurodegeneration in MPTP-treated mice and PD patients. To dissect the mechanism of miR-425 action, we validate that reduced miR-425 promotes necroptosis by targeting the 3′UTR of RIPK1. Next, in a miR-425 knockdown mouse model, we demonstrate that miR-425 inhibition induces the upregulation of RIPK1 and necroptosis activation. From a therapeutic perspective, our current results suggest that miR-425 supplements in dopaminergic neurons could reduce necroptosis and may be a valid therapeutic approach for PD. Alternatively, it could be combined with other therapeutics that aim to block the neurotoxic insult, especially MPTP.

MPTP is a neurotoxin that recapitulates the neuropathology of PD and causes specific loss of dopaminergic neurons in animals and a profound reduction of striatal dopamine levels^35^. MPTP could be specifically uptaken by dopaminergic neurons and targets the mitochondria of neurons^36^. Neuronal degeneration is caused by its toxic metabolite MPP^+^, followed by mitochondrial dysfunction induced by elevated oxidative stress^25^. How this toxicity induces intracellular protein changes and mediates cell death remains ambiguous. Our results show that MPTP could regulate posttranslational modification of necroptosis-associated gene through miR-425. Furthermore, we highlight the role of miR-425-RIPK1 axis in mediating the inflammatory responses and neuronal death. Previous reports suggest that RIPK1 activation results in the regulation of ROS, mitophagy, and oxidative stress^11,27,37^. Consistent with those reports, our findings further reveal that miR-425-mediated RIPK1 regulation serves a critical role in dopaminergic neurodegeneration.

According to previous studies and our results, MPTP treatment induces prominent dopaminergic neuron loss and dopamine depletion in the striatum and the SNpc with impaired dopaminergic system functions^23,38^. This dysfunction and pathology were aggravated by miR-425 knockdown in the MPTP-treated mice model. MPTP neurotoxicity in vivo is thought to arise from superoxide production due to mitochondrial complex I inhibition accompanied by microglial activation and inflammatory response^35,39^. Our results show that miR-425 inhibition could also increase both ROS production and mitochondrial disruption, which correlates with the occurrence of dopaminergic neuron loss and dopamine depletion. These findings bridge the relationship between oxidative stress and necroptotic neuron death in the pathogenesis of PD^40^.

When finishing the experiments in MPTP-treated mice, we cannot neglect an unavoidable problem: Since MPTP-treated mouse is an artificial PD model, how about the miR-425 and RIPK1 levels in human brain tissue? Thus, we validate the role of miR-425 in neurodegeneration and neuron death in SN sections from patients with PD and controls. We find that that brain enriched miR-425 is markedly reduced in SN of PD brains, and a higher degree of colocalization between miR-425 and dopaminergic neurons. Moreover, the necroptosis-associated proteins, including RIPK1, RIPK3, and pMLKL, are significantly increased in PD cases compared with the control. Therefore, dopaminergic neurodegeneration by triggering necroptosis activation in SN of PD patients are successfully validated for the first time so far. Our data broaden the spectrum of molecular pathogenesis of PD from bench to bed.

However, this study does not address the detailed mechanism of MPTP-mediated downregulation of miR-425 expression. A possible explanation is that the inflammatory response cytokines such as TNFα might mediate transcriptional activity suppression by miR-425. Meanwhile, we show that decreased levels of miR-425 result in higher activation of the necroptosis pathway. Increased necroptosis and disruption of the cell membrane may promote miR-425 degradation. Then, a vicious circle between necroptosis and miR-25 would occur.

## Conclusion

In conclusion, our data indicate that miR-425 deficiency in PD triggers necroptosis of dopaminergic neurons, and targeting miR-425 in MPTP-treated mice restored dysfunctional dopaminergic neurodegeneration and ameliorated behavioral deficits. Our results establish a previously undescribed link between RIPK1 and miR-425 and propose miR-425 supplements as a probable therapeutic approach for neurodegenerative disease with neuron loss.

## Supplementary information

Supplementary files

Tabble S1
