# Supplementary material for: miR-425 deficiency promotes necroptosis and dopaminergic neurodegeneration in Parkinson’s disease
Source: Cell Death Dis. 2019 Aug 5;10(8):589. doi: 10.1038/s41419-019-1809-5 (PMC6683159; doi:10.1038/s41419-019-1809-5)
Supplement: Supplementary file 1 — Supplementary files [file 41419_2019_1809_MOESM1_ESM.docx]

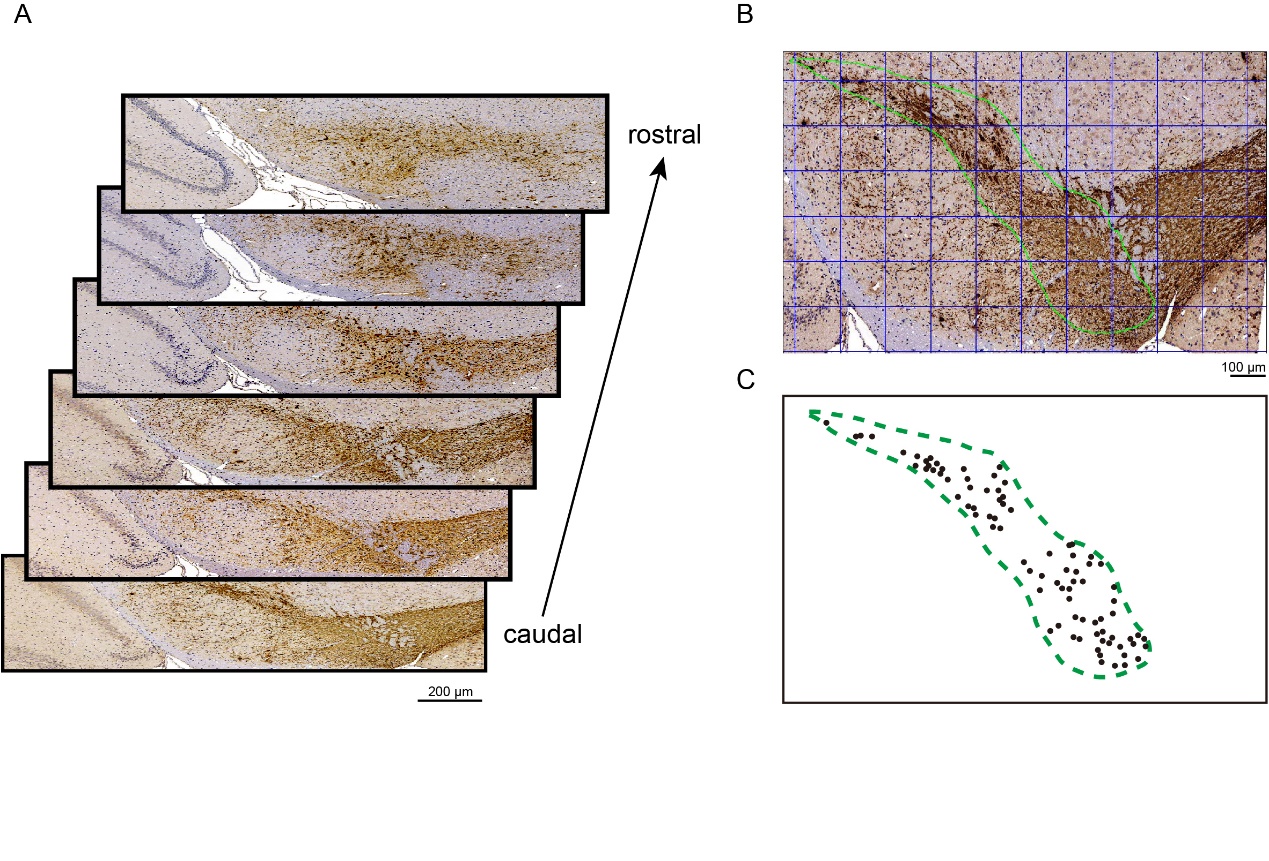


**Figure S1 Processing the images for stereological estimation.**

(A) A series of TH-stained substantia nigra sections covered the mouse substantia nigra from rostral to caudal;

(B) An example stack image is taken from a mouse substantia nigra. After the randomized insertion of a grid overlying the SNpc, the SNpc was outlined (green line);

(C) The TH-positive neurons in SNpc were depicted.


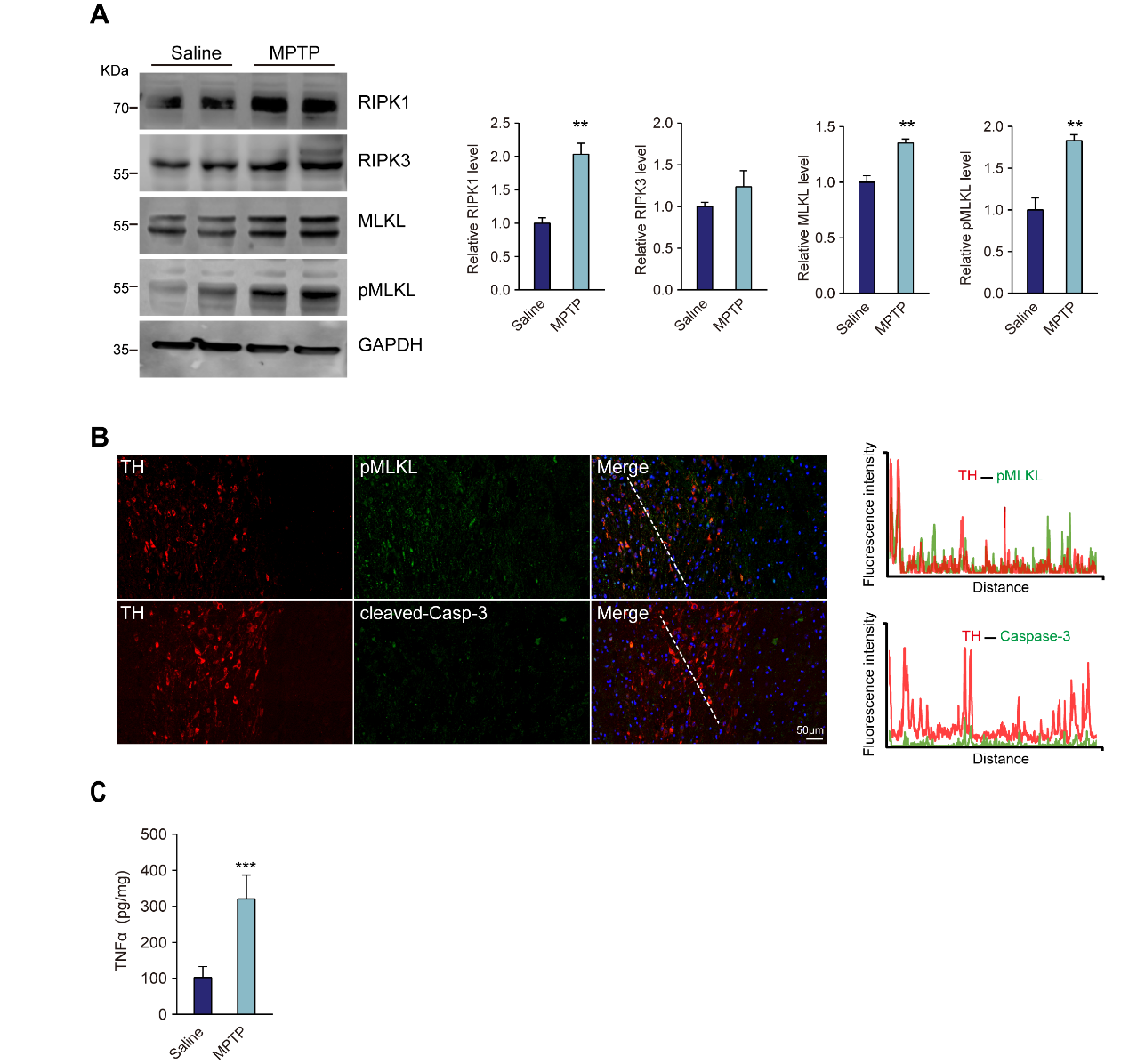


**Figure S2 Necroptosis activation in dopaminergic neurons of MPTP-treated mice.**

A. Immunoblotting and quantification of RIPK1, RIPK3, MLKL and pMLKL expression in the midbrain SNpc of MPTP- and saline-treated mice.

B. Immunohistochemistry and colocalization for necroptotic marker pMLKL and apoptotic marker cleaved Caspase-3 with TH-positive dopaminergic neurons in the SNpc of MPTP-treated mice.

C. Quantification of TNFα in the SNpc.

All data were represented as mean ± SEM. Student’s t-test, **P < 0.01, ***P < 0.001.

**Table S1 Predicted miRNAs binding the 3’UTR of RIPK1.**

**
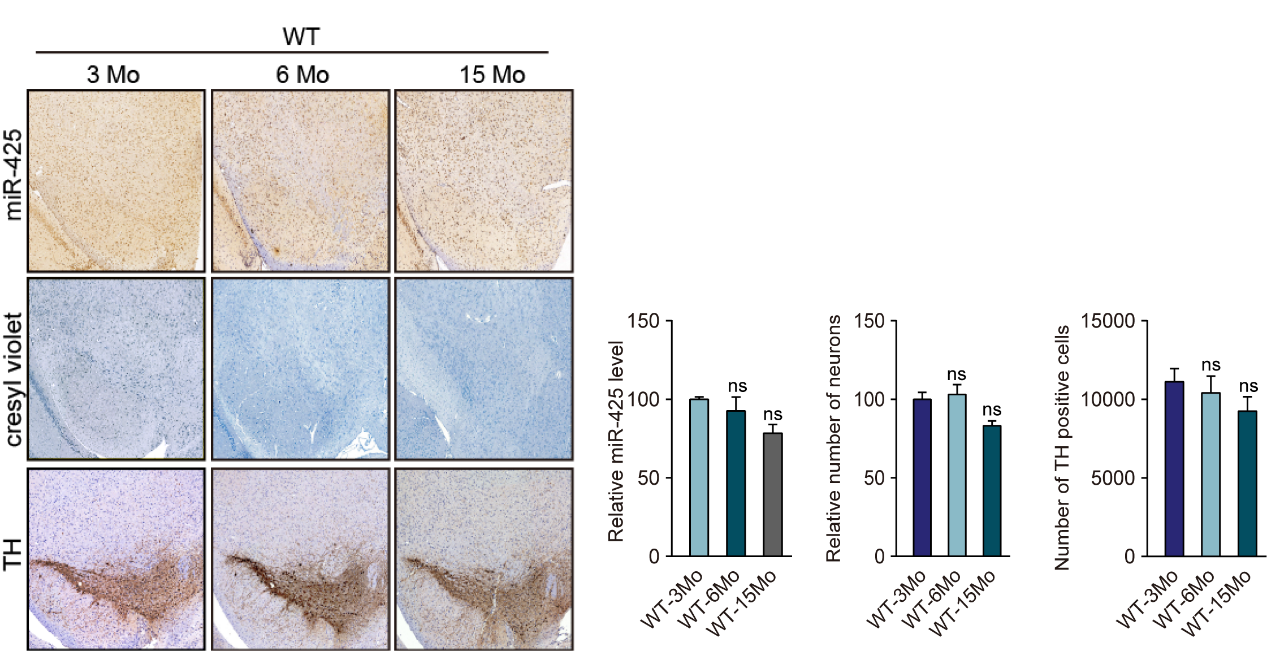
**

**Figure S3 Age-dependent changes in SNpc of Mir-425 WT mice.**

Chromogenic ISH of miR-425, immunohistochemistry for TH and cresyl violet-positive cells in miR-425 WT mice at 3 months (3 Mo), 6 months (6 Mo) and 15 months (15 Mo).

All data were represented as mean ± SEM. One-way ANOVA followed by Dunnett’s post hoc test was applied, ns, not significant.
